# Interpreting mutual adjustment for multiple indicators of socioeconomic position without committing mutual adjustment fallacies

**DOI:** 10.1186/s12889-018-6364-y

**Published:** 2019-01-03

**Authors:** Michael J. Green, Frank Popham

**Affiliations:** MRC/CSO Social & Public Health Sciences Unit, 200 Renfield Street, Glasgow, G2 3AX UK

**Keywords:** Socioeconomic position, Education, Occupation, Income, Regression, DAGs, Causal inference

## Abstract

Research into the effects of Socioeconomic Position (SEP) on health will sometimes compare effects from multiple, different measures of SEP in “mutually adjusted” regression models. Interpreting each effect estimate from such models equivalently as the “independent” effect of each measure may be misleading, a mutual adjustment (or Table 2) fallacy. We use directed acyclic graphs (DAGs) to explain how interpretation of such models rests on assumptions about the causal relationships between those various SEP measures. We use an example DAG whereby education leads to occupation and both determine income, and explain implications for the interpretation of mutually adjusted coefficients for these three SEP indicators. Under this DAG, the mutually adjusted coefficient for education will represent the direct effect of education, not mediated via occupation or income. The coefficient for occupation represents the direct effect of occupation, not mediated via income, or confounded by education. The coefficient for income represents the effect of income, after adjusting for confounding by education and occupation. Direct comparisons of mutually adjusted coefficients are not comparing like with like. A theoretical understanding of how SEP measures relate to each other can influence conclusions as to which measures of SEP are most important. Additionally, in some situations adjustment for confounding from more distal SEP measures (like education and occupation) may be sufficient to block unmeasured socioeconomic confounding, allowing for greater causal confidence in adjusted effect estimates for more proximal measures of SEP (like income).

## Background

Socioeconomic position (SEP) can be defined as the “the social and economic factors that influence what position individuals or groups hold within the structure of a society” [1] and is widely recognised as associated with health [2, 3]. Understanding causal effects of SEP on health can be problematic as SEP is a broad and heterogeneous concept, difficult to operationalise [1, 4], and it may act via a multitude of mechanisms or pathways [2]. Various SEP measures are often used interchangeably despite differences in their theoretical grounding and interpretation, and without regard to the level (e.g. individual, household, area) or life-stage at which they are measured [1, 4–9]. Nevertheless, researchers sometimes try to differentiate the importance of different SEP indicators. While a range of more advanced methods have developed in recent years that could be employed in this context, we have noticed that many researchers still default to mutually-adjusted regression models of health, which require careful interpretation. Regression models may be seen as ‘good enough’, while more advanced methods are seen as offering diminishing returns to effort. With this in mind, the aim of this article is to highlight potential interpretive pitfalls when mutually adjusting for multiple measures of SEP, explaining best practice and the limitations and assumptions that are implicit with such models. Using directed acyclic graphs (DAGs), we emphasise that causal interpretations of such analyses are based on assumptions about causal relationships between SEP measures.

### Multi-dimensionality of SEP

**Fig. 1:**
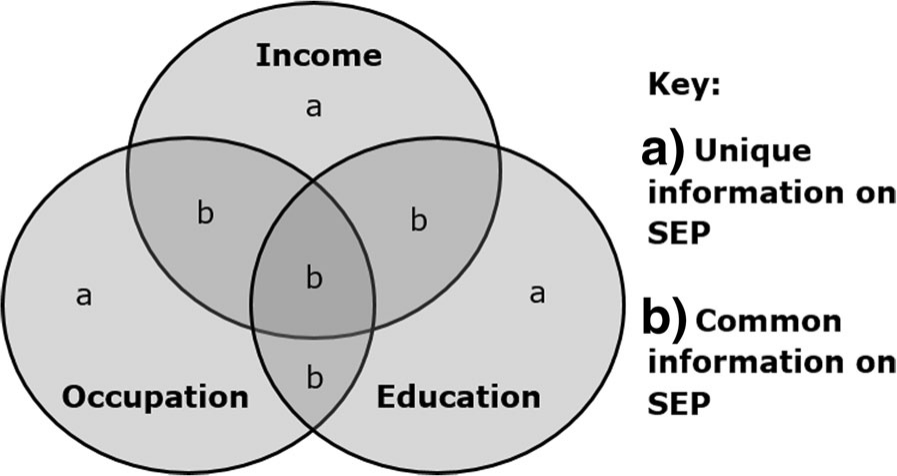
Common and unique representation of SEP by income, occupation and education

One barrier to understanding socioeconomic inequalities in health is that SEP represents more than just one thing. It comprises multiple dimensions, including economic or material resources, social status or prestige and political power [4, 7, 10, 11], and a person may be advantaged in some respects but not others [7, 11, 12]. SEP measures may each represent the general concept to some degree, but each may be considered to represent some common and some unique information (as illustrated for education, occupation and income in Fig. 1) [1, 13]. Where measures overlap in capturing general social and economic standing [1, 4], this shared ‘core’ of SEP is the areas labelled ‘b’. Nevertheless, different measures may be especially indicative of particular resources: the areas labelled ‘a’. For example, occupations may be particularly indicative of working conditions [1, 4], while education is especially indicative of knowledge and skills [1, 4, 14], and income specifically represents acute access to material resources [5, 15]. This pattern of some overlapping and some specific information is not unique to income, occupation and education, but could be applied to almost any set of SEP measures, including measures of the same indicator at different life-stages (e.g. income at ages 15, 45 and 75) or measured at different levels (e.g. unemployment at the individual, household and area level) [1, 5, 9].

SEP measures are often correlated but could rarely be treated as proxies for each other [7]. Where SEP is viewed as a confounder for effects of other exposures, this multi-dimensionality may be a minor nuisance: adjustment for a single measure of SEP may leave residual confounding, and best practice would include adjustment for multiple (or all available) measures of SEP [7, 16]. At least this is true as long as SEP is really a confounder and not a mediator of the exposure, if any of the SEP measures could be considered mediators of the exposure effects then adjusting for these could induce collider bias via unobserved SEP [17, 18]. However, when SEP is the causal exposure of interest this multi-dimensionality is more critical, and may even be informative.

Consistent associations with health across a range of SEP measures suggest a relationship between the overall ‘core’ construct of SEP and health. Some even investigate this ‘core’ effect of SEP by aggregating information from multiple SEP measures, e.g. as a latent variable [19], or index counting disadvantages experienced [20, 21].

However, investigation of heterogeneity across multiple measures may help identify which characteristics or resources associated with SEP are most important for health, and which factors could best be intervened on in order to alleviate health inequalities [9, 16, 22, 23]. For example, one study found inequalities in a range of health outcomes were stronger for occupational class when stratifying by education than vice versa, concluding adult occupational class was “a better discriminator of socioeconomic differentials” [24]. Authors of a US study showing clearer associations with mortality for income than for education and occupation argued for the systematic collection of administrative income data for the monitoring and analysis of health inequalities [15]. A Finnish study finding independent associations with smoking for a range of socioeconomic variables concluded that alleviation of inequalities in smoking requires “efforts directed at various dimensions of socioeconomic position” [13]. Numerous studies have compared SEP measures across the lifecourse to infer regarding the lifecourse mechanisms leading to health inequalities [16, 22, 24, 25].

Certainly, more advanced methods are becoming more popular for addressing such issues, with examples including: applications of machine-learning methods to identify the most important predictors of health outcomes [26], path analyses or structural equation models to test hypotheses regarding lifecourse pathways [19, 27], and Bayesian approaches where observed data are used to indicate the most likely causal structure linking a set of variables [28, 29]. Nevertheless, many studies have used regression-based models where SEP measures are mutually adjusted [13, 15, 16, 22, 24, 25, 30, 31], and it is not hard to find even very recent examples e.g. see [32–35]. We focus therefore on explaining best practice for such models, highlighting potential interpretative pitfalls and their implicit assumptions and limitations.

### The “mutual adjustment” fallacy

Interpretation of mutually adjusted regression models requires care and conceptual clarity (as exemplified in many of the citations given above). It is tempting to interpret the mutually adjusted regression coefficients for each SEP measure as the independent effects of the unique aspects of SEP represented by that measure (i.e. the effects of the areas labelled ‘a*’* in Fig. 1). However, if this interpretation is carried across all the included measures of SEP, then aspects of SEP that are common across measures (i.e. the areas labelled ‘b’ in Fig. 1) have been assumed to have no effect. With only three coefficients and each interpreted as the effect of its respective ‘a’ area, the overlapping information in ‘b’ is not represented by a coefficient. Worryingly, if this area of conceptual overlap represents the ‘core’ of the SEP construct, then this assumes ‘core SEP’ has no effect and that all effects are due to the unique characteristics of the different SEP measures employed.

This can be considered a case of the “Table 2” fallacy, where mutually adjusted coefficients are treated as if they all have an equivalent interpretation [36]. The idea was so named because mutually adjusted coefficients have often been presented in the second results table of a paper. We prefer the term “mutual adjustment” fallacy as this is a little more explicit about the error being committed, which could occur in the first, third, fourth or any other results table.

### Causal interpretation of a mutually-adjusted model

**Fig. 2:**
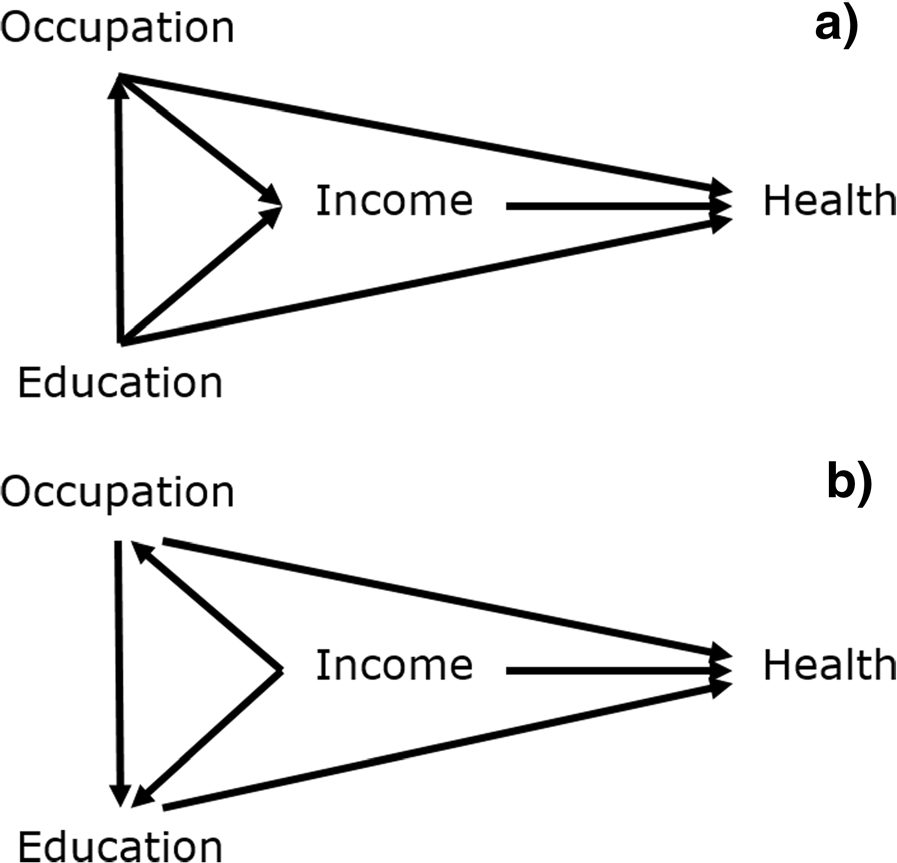
**a**: a plausible causal diagram; **b**: an alternative causal diagram

Here we use causal diagrams (or DAGs [37]) to interpret mutually-adjusted coefficients under specific assumptions about the causal relationships between variables [23, 36]. Consider Fig. 2a, which is a plausible causal diagram of health and three measures of SEP: education, occupation and income (with all three measures taken in middle-age). Arrows represent causal effects, with all SEP measures having a causal effect on health. There are also causal relationships between the SEP measures, with income viewed as the most proximate cause, and education the most distal. The effect of education is both direct and mediated via occupation and income; the effect of occupation is both direct and mediated via income but confounded by education; and the effect of income is confounded by occupation and education. Figure 2a also assumes no further unmeasured confounding (we return to unmeasured confounding later). Such a situation might be investigated using a mutually-adjusted regression model of health on education, occupation and income, but the coefficients or odds ratios (ORs) associated with these three SEP measures represent different causal effects [36].

Table 1 illustrates with data taken from a baseline survey of 35 year-olds in the West of Scotland: Twenty-07 Study (*n* = 1248) [38]. Data were coded to indicate low education (left school at age 16 or earlier), manual (compared to non-manual) occupations [39], and low income (lowest tertile of equivalised household income). Self-assessments of health as poor (vs. excellent through fair) were taken as the outcome (5.9% reported poor health). The first column of the table shows unadjusted odds ratios (ORs) and 95% confidence intervals (95% CI) for the association between each measure of SEP and self-assessed health. Other columns show models with adjustment for occupation and education only, and with mutual adjustment for all three measures of SEP. As the outcome is rare we will ignore issues related to the non-collapsibility of ORs [40].

**Table 1:** Illustrative example of mutual adjustment

	Unadjusted ORs for poor health (95% CI)	Interpretation under Fig. 2a	Partially Adjusted ORs for poor health^a^ (95% CI)	Interpretation under Fig. 2a	Mutually Adjusted ORs for poor health^b^ (95% CI)	Interpretation under Fig. 2a
Low Education	2.43 (1.32–4.47)	Total effect of education	1.64 (0.84–3.19)	Direct effect of education, not mediated via occupation	1.44 (0.72–2.85)	Direct effect of education, not mediated via occupation and income.
Manual Occupation	2.79 (1.73–4.48)	Total effect of occupation, confounded by education	2.33 (1.39–3.93)	Total effect of occupation, not confounded by education	1.99 (1.15–3.43)	Direct effect of occupation, not mediated via income, or confounded by education.
Low Income	2.48 (1.55–3.98)	Total effect of income, confounded by education and occupation	–		1.68 (0.99–2.85)	Total effect of income, not confounded by occupation and education.

^a^Education and Occupation only

^b^Education, Occupation and Income

Under Fig. 2a, the mutually adjusted OR for income represents the total effect of income on health, after adjusting for confounding by occupation and education [36]. However, the mutually adjusted OR for occupation does not have the same interpretation; it is not a total effect of occupation, but rather a direct effect of occupation, i.e. the portion of its total effect on health which is not mediated via income, after adjusting for confounding by education [36]. Similarly, the mutually adjusted OR for education represents a direct effect, not mediated via occupation or income [36]. Thus, a comparison of the mutually adjusted ORs for education and income is not a like-for-like comparison, but a comparison of a total effect (for income) with a direct effect (for education). If the intent was to compare the total effects of education and income then this would be an incorrect comparison: a mutual adjustment fallacy.

Given Fig. 2a, a question about the relative magnitude of the total effects of these SEP measures could be answered by comparing the mutually adjusted OR for income (1.68), with the partially adjusted OR for occupation (2.33), and the unadjusted OR for education (2.43), i.e. adjusting for confounders but not mediators. This highlights quite a different pattern in the magnitude of effects as would be obtained by straight comparison of the mutually adjusted (or the unadjusted) ORs.

### Competing assumptions

Figure 2a is of course not the only set of assumptions that could be made about the relationships between these variables, and analyses may be sensitive to different assumptions [23]. Figure 2b shows an alternative set of (less plausible) causal assumptions to those shown in Fig. 2a. In this case, education is now considered the more proximate and income the more distal cause. Under Fig. 2b, a question about the relative magnitude of the total effects of education and income would require comparison of the mutually adjusted OR for education with the unadjusted OR for income (i.e. 1.44 and 2.48 respectively in Table 1). Thus, different assumptions about the causal relationships between socioeconomic variables can lead to different conclusions about which are more important.

If Fig. 2a and b were both equally plausible, one might want to compare total effect sizes under competing causal assumptions: e.g. the total effect of education with income and occupation viewed as confounders, vs. the total effect of income with education and occupation viewed as confounders. In this case, a comparison of the mutually adjusted ORs for education and income would be appropriate. However, you would only want to do this in situations with multiple plausible competing causal assumptions. In such cases, interpretation could be facilitated by clear description of the competing assumptions, explicitly linking the reported results to those assumptions. We consider ‘mutual adjustment’ to be poor short-hand for such a nuanced interpretation.

Further, we would suggest the causal ordering in Fig. 2a rather than Fig. 2b is more plausible and more consistent with theory regarding the development of SEP over the lifecourse, with education usually preceding and determining occupational level, which in turn generates income. [10, 41] However, while we would favour interpretation under Fig. 2a over interpretation under Fig. 2b, it would be poor practice to assume all other researchers share our assumptions. Reporting the findings in Table 1 as mutually adjusted without explicitly linking them to the assumptions in Fig. 2a could lead to misinterpretation. A naïve reader would have no guidance as to which effects should be interpreted as total and which as direct effects, or a reader who strongly subscribed to Fig. 2b might interpret the results according to those assumptions. Phrases such as “the independent effect of occupation” do not specify whether the “independence” is from confounders or mediators and are thus less informative than they could be with further explication.

### Unmeasured confounding

**Fig. 3:**
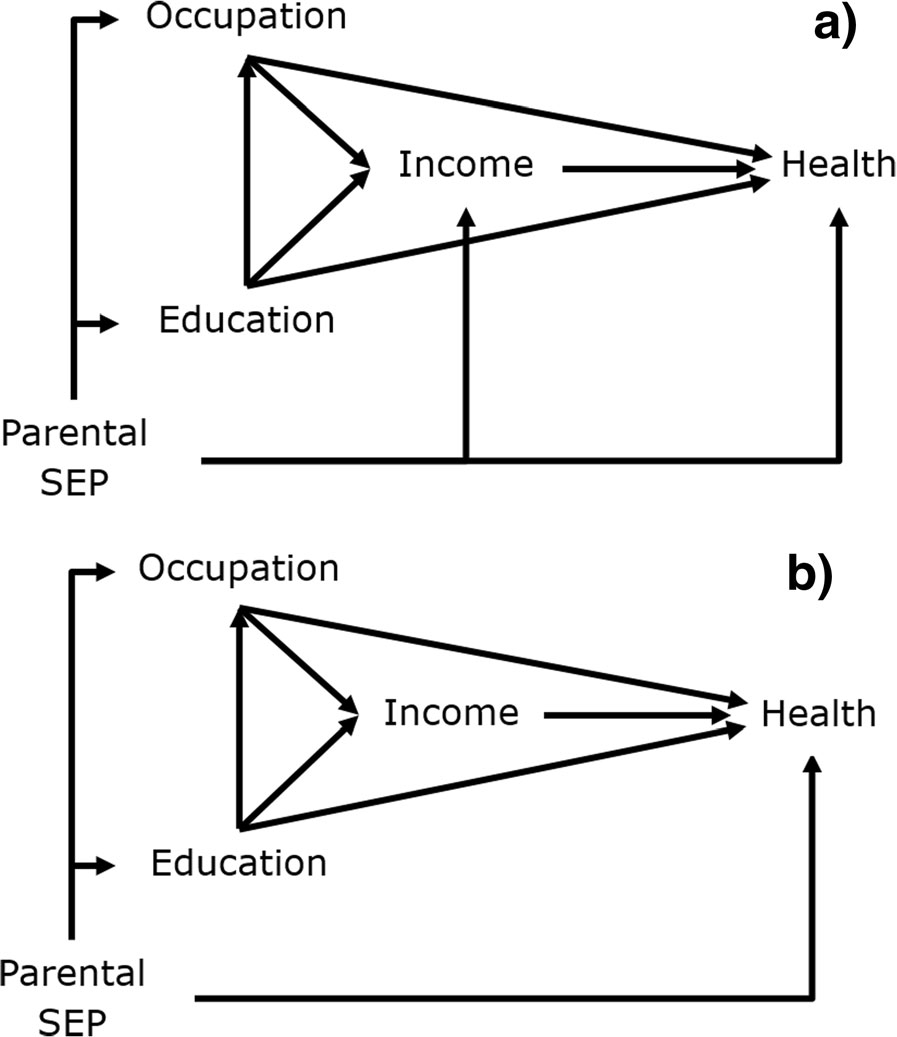
**a**: Full unmeasured confounding from Parental SEP; **b**: Partial unmeasured confounding from Parental SEP

Given how broad the concept of SEP is, there are likely to be aspects of SEP not fully captured by any particular set of measures used [23, 35]. Thus, it is important to consider how interpretations might be affected by unmeasured socioeconomic information (or other unmeasured confounders, which would affect interpretation in a similar way). Consider the two alternative situations in Fig. 3, which re-create Fig. 2a but add parental SEP as a distal, antecedent cause of health and other socioeconomic variables. In Fig. 3a parental SEP is assumed to be a determinant of health, education, occupation and income, whereas in Fig. 3b it determines health, education and occupation only, and is otherwise independent of income. Under Fig. 3a, if parental SEP were unmeasured then all the effect estimates (of both total and direct effects) derived from the mutually adjusted model in Table 1 are potentially biased. This is due to the confounding influence of parental SEP, though the direct effects of education and occupation could also be biased due to having conditioned on a collider [17, 18].

However, under Fig. 3b the estimate of the total effect of income from the mutually adjusted model in Table 1 remains unbiased. The confounding influence of unmeasured parental SEP on the effect of income is sufficiently blocked by adjustment for own education and occupation, though the direct effects of occupation and education are still subject to bias from the confounding influence of unmeasured parental SEP and from conditioning on a collider [17, 18]. Thus, if Fig. 3b were at all plausible relative to Fig. 3a we might have more confidence in the total effect estimate for income from Table 1 than in the direct effect estimates for occupation and education. This total effect estimate requires less stringent assumptions to be valid (i.e. Figure 3b) than those for the direct effect estimates (i.e. Figure 2 with no unmeasured confounding).

We illustrate further with an indicator of parental SEP (manual vs non-manual occupation of parents at age 15) in the T07 data, and empirically test whether Fig. 3a or b is more realistic. Table 2 takes low income instead of poor health as the outcome and shows regressions of this on parental occupation, with and without adjustment for own education and occupation. While parental occupation is associated with low income with an OR of 2.80 (CI: 2.08–3.77), adjustment for own education and occupation substantially attenuates this association to 1.28 (CI: 0.91–1.80). Thus, in this situation given own education and occupation, low income at age 35 is relatively independent of parental occupation, and Fig. 3b is a plausible alternative to Fig. 3a. Adjustment for own education and occupation would probably be sufficient to estimate effects of income on health among these data, without confounding bias from parental occupation. Thus, adjustment for some distal SEP measures can plausibly block confounding from unmeasured (and more distal) aspects of SEP.

**Table 2:** Logistic regression predicting low income from parental occupation, with and without adjustment for own education and occupation

	Unadjusted OR for Low Income (95% CI)	Adjusted OR for Low Income (95% CI)
Manual Parental Occupation	2.80 (2.08–3.77)	1.28 (0.91–1.80)
Low Education	–	4.66 (3.08–7.05)
Manual Occupation	–	3.83 (2.89–5.08)

## Discussion

We focused here on the relatively simple method of mutually adjusted regression, without tackling issues such as interactions between SEP measures, effect heterogeneity, measurement error or statistical mis-specification [23]. As mutually adjusted regression is still commonly used in epidemiology, we hope this discussion will aid best practice, highlight implicit assumptions and limitations, and provide an introductory step towards the ever-developing literature on more advanced causal inference methods, which might include Bayesian analyses [28, 29] or more precisely decomposed definitions of direct and indirect effects that can deal appropriately with interactions between SEP measures [42–46].

We examined four possible causal diagrams linking three SEP measures, but many more are possible [23], and interpretations of analyses should be altered under differing causal assumptions, as illustrated here. Indeed, the causal structure linking SEP indicators to each other and to health may vary between different social contexts, welfare regimes etc. [28], as may the degree of heterogeneity both within and between socioeconomic categories [9], so researchers may be unsure of what causal model to interpret their data under. Causal direction may also be ambiguous where there are feedback loops (e.g. where a better job leads to a higher income, which then leads to an even better job and so forth). In some cases, such longitudinal ambiguity may be resolved by including repeated measures from different life-stages and interpreting analyses in light of the causal links between measures over time [47], but researchers will not always have the luxury of such detailed lifecourse data. Where there is ambiguity over the causal structure linking socioeconomic variables, Bayesian methods offer a promising avenue for empirically determining the most likely structure given the data [28, 29]. Indeed, such exploratory research is important for developing a good understanding of the causal relationship between socioeconomic variables. Nevertheless, data-driven approaches such as these may be sensitive to idiosyncrasies of the data under study, and/or there may be multiple causal diagrams that would fit the data more or less equally well. Thus, it may still be worthwhile to assess how sensitive analytical conclusions are to alternative theoretically informed causal diagrams.

We highlight the importance of a full causal diagram that includes all relevant common causes of included variables, which might include distal and unmeasured aspects of SEP [23]. Given difficulties associated with gathering inter-generational socioeconomic data, our illustration of residual confounding from parental SEP is a pertinent one. It demonstrates how, in some instances, adjustment for some socioeconomic confounders may be sufficient to block confounding from other more distal and unmeasured aspects of socioeconomic background, and give greater confidence that effect estimates from a proximal measure of SEP are causal. On the other hand, we have shown how estimates of direct effects for more distal measures of SEP can be biased by residual unmeasured confounding [35], and/or collider bias [17]. Where there is the likelihood of residual unmeasured confounding (i.e. almost always) it would seem advisable to test sensitivity of conclusions to different strengths of unmeasured confounding [48].

Estimating “effects” of SEP measures presupposes intention to intervene on and manipulate SEP [23, 46]. Assuming Fig. 2a to be the more plausible set of assumptions regarding the causal relationships between education, occupation and income (which may not hold in all contexts [28]), it follows that we could have greater causal confidence in published estimates of the effects of income that are adjusted for education and occupation (or other more distal socioeconomic variables), than we might have in published estimates of the effects of occupation or education (whether or not they are adjusted for other socioeconomic measures). This is fortuitous, as of the three, it could be argued that income would also be the simplest to intervene on [49], e.g. through welfare or taxation policies. Estimates of effects for education or occupation that are adjusted for income are not without their merit however, and, assuming no unmeasured confounding, could be interpreted for example as the remaining effect of occupation, if we were to somehow intervene and equalise income across occupational strata [46].

## Conclusion

The principles set forth here are not limited to applications involving education, occupation and income but will be applicable whenever researchers are using mutual regression adjustment to compare the effects of different SEP measures, including measures taken at different levels or life-stages. We have explained how effects for multiple socioeconomic measures should not be interpreted equivalently as the “independent” effect of that measure, ignoring the effects of the common information they provide. Causal effects of more distal SEP measures, will be biased with adjustment for proximate SEP measures. A good causal understanding of relationships between socioeconomic variables can improve interpretation and lead to different conclusions about which measure is most important for health than naïve comparisons of “independent” effects. Such understanding is therefore foundational to understanding the causal processes by which SEP influences health over the lifecourse, and to identifying effective points of intervention [23].

## Abbreviations

CI: Confidence Interval
DAG: Directed Acyclic Graph
OR: Odds Ratio
SEP: Socioeconomic Position
